# Nutrition Value of Baked Meat Products Fortified with Lyophilized Dragon Fruit (*Hylocereus undatus*)

**DOI:** 10.3390/foods12193550

**Published:** 2023-09-24

**Authors:** Paulina Kęska, Patrycja Gazda, Łukasz Siłka, Katarzyna Mazurek, Joanna Stadnik

**Affiliations:** Department of Animal Food Technology, Faculty of Food Science and Biotechnology, University of Life Sciences in Lublin, Skromna 8, 20-704 Lublin, Poland; paulina.keska@up.lublin.pl (P.K.);

**Keywords:** functional meat product, dragon fruit, pitaya, pork, lyophilized

## Abstract

This study evaluates the nutritional value of a baked pork meat product containing lyophilized dragon fruit pulp. The selected nutritional properties of a baked pork meat product fortified with lyophilized *Hylocereus undatus* pulp in doses of 0.5%, 1.5%, 2.5%, and 4% were evaluated. For this assessment, changes in the basic chemical composition of the products, the content of calcium, magnesium, potassium, iron, and phosphorus, and the profile of fatty acids were considered. Additionally, characteristics typical for meat products, such as pH, water activity, oxidation-reduction potential or thiobarbituric acid reactive substances, and antioxidant properties of the product during 21 days of refrigerated storage, were assessed. The findings indicate that the use of higher doses of lyophilizate, i.e., in the amounts of 2.5% and 4%, significantly (*p* < 0.05) increases the nutritional value of meat products, leading to an increase in the concentration of essential minerals important for the proper functioning of the human body (calcium, magnesium, potassium, and iron). These changes occurred without affecting the basic chemical composition (except for an increase in the content of fat and carbohydrates in the sample with the addition of 4% lyophilizate). The introduction of the fortification treatment improved the fatty acid profile, resulting in an increase in the content of C14:0, C16:0, C20:0, and C20:5n3. In addition, in the variant with a 4% dosage, there was an increased content of C8:0, C10:0, C16:1n7, C18:0, C18:1n9C + C18:1n9t, and C18:2n6C + C18:2n6t, C18:3n3 (alpha), C20:1n15, and C20:1n9. In this particular variant, an increase in saturated-, monounsaturated-, and polyunsaturated fatty acids was also observed, which was associated with an increased level of TBARS in meat products. However, the increase in the dose of lyophilizate caused an increase in the antiradical effect of meat extracts. Based on the results obtained, it seems reasonable to use a plant additive in the form of lyophilized dragon fruit pulp in the amount of 4.0% in the production of pork meat products.

## 1. Introduction

Plants are rich in bioactive compounds (BAC), mainly polyphenols, which are the most extensively researched group of compounds recognized for their strong antioxidant properties in the scientific literature. Repeated studies have consistently shown that a vegetable supplement rich in polyphenols can be a good alternative to synthetic antioxidants in meat products. This approach aims to preserve the quality of meat products with similar or better technological attributes compared to synthetic food additives. Their inclusion in fresh meat products can effectively maintain oxidative stability, stabilize color parameters as well as lipid and protein oxidation, and limit the use of nitrogen compounds without affecting the sensory properties or storage stability [1,2]. In addition, these new, bioactive plant-derived compounds contribute significantly to improving the nutritional and functional qualities of the fortified product, thereby diversifying the range of offerings in the domain of “wellness foods.”

Dragon fruit (pitaya) is a tropical fruit belonging to the *Cactaceae* family, genus *Hylocereus*. There are mainly three commercial varieties distinguished by the color of their skin and flesh: *Hylocereus undatus* (red skin and white flesh), *Hylocereus polyrhizus* (red skin and red flesh), and *Hylocereus megalanthus* (yellow skin and white flesh) [3]. Dragon fruit is considered one of the “superfoods” due to its richness in nutrients and relatively low caloric content. The bioactive compounds contained in this fruit include antioxidants (ascorbic acid, tocopherol, and polyphenols), fiber, vitamins, and minerals like magnesium, calcium, phosphorus, and potassium, along with betacyanin, p-coumaric acid, protocatechuic acid, vanillic acid, gallic acid, syringic acid, and p-hydroxybenzoic acid [4,5,6,7,8]. The content of individual components may vary depending on the plant variety and the edible parts obtained from it. As an example, studies have shown a higher proportion of polyphenols in the fruit peel extracts of the *H. polyrhizus* cultivar than in the case of *H. undatus*. In addition, the fruit peel exhibits potential as an antibacterial and antioxidant agent, mainly due to the presence of betacyanins, and can also serve as a natural dye or source of pectins. Choo et al. [6] studied the antioxidant properties of fruits (peel and flesh) from two species, *H. polyrhizus* and *H. undatus*. Their findings showed that the content of ascorbic acid and total phenols in pulps was higher than in the whole fruits (peel and pulp) of both Hylocereus species. At the same time, they indicated a greater role for ascorbic acid (over phenols) while presenting the antioxidant properties of the plant. The authors analyzed the antiradical activity of extracts obtained from plant material as measured by DPPH radical scavenging activity and ranked them in the following order: *H. undatus* flesh ≈ *H. polyrhizus* flesh > fruit (peel and flesh) *H. polyrhizus* > fruit (peel and flesh) *H. undatus*. Similarly, the ability to chelate iron ions was ranked as follows: *H. undatus* flesh > fruit (peel and pulp) *H. polyrhizus* > fruit (peel and flesh) *H. undatus* > flesh of *H. polyrhizus* [6]. Furthermore, dragon fruit seeds are an excellent source of essential unsaturated fatty acids, often used for oil extraction [5,7,8]. Due to the abundance of BAC, fruits of the genus *Hylocereus* have the potential to prevent diseases caused by oxidative factors or reduce the effects of inflammatory processes. As a result, dragon fruit may have several benefits for conditions such as diabetes, dyslipidemia, metabolic syndrome, cardiovascular disease, and cancer. It also exhibits antimicrobial activity [5,9]. Tamagno et al. [10] suggest supplementation with microencapsulated pitaya (*H. undatus*) pulp extract to reduce the effects of copper poisoning and prevent metal-induced oxidative damage. Given the abundance of various BACs and their versatile effects, the use of dragon fruit to enrich popular food types seems to be an interesting alternative, especially considering reports that have shown that dragon fruit extracts at doses ranging from 1250 to 5000 mg/kg and their consumption have not shown toxicity and did not cause disturbances or abnormalities in animal organ systems [5,11].

Recent reports in the literature have indicated the possibility of using dragon fruit to enrich animal-based foods. For instance, dragon fruit has been added to milk products [12,13,14] as well as chicken [15,16,17], beef [18], pork [19,20,21], and mutton [22], serving various roles such as reducing fat oxidation, offering antibacterial properties, and enhancing color. The addition of *Hylocereus* spp. to food items may also have the additional effect of improving consumer health by enriching them with essential components like vitamins, minerals, fatty acids, fiber, and natural antioxidants. This enrichment can occur without the need for additional supplementation beyond the normal daily diet.

In this study, the nutritional value of the meat product with the addition of lyophilized dragon fruit pulp was assessed. Among various aspects, the proximate composition, selected minerals (such as calcium, iron, potassium, magnesium, and phosphorus), and the fatty acid profile of the products were assessed. Additionally, the antioxidant potential of the products was also assessed by evaluating their antiradical effects (using ABTS^•+^ and DPPH radicals) and reduction power (RP). For a better interpretation of the results, parameters related to the quality of meat products, such as pH, aw, ORP, and TBARS, were assessed.

## 2. Material and Methods

### 2.1. Preparation of Dragon Fruit Pulp and Extract

The dragon fruit (*H. undatus*) was procured from a local store, thoroughly washed under warm running water, and then peeled. The resulting pulp (with seeds) was then frozen and lyophilized. Freeze-drying was carried out for 72 h using a freeze dryer (Free Zone 12 lyophilizer, Labconco Corporation, Kansas City, MO, USA) set at −80 °C and 0.04 mbar. The resultant powder was carefully stored in airtight containers at room temperature for further use. Before use, the lyophilizates were ground in a colloid mill to a powder. In order to assess the antioxidant potential of the lyophilized *H. undatus* pulp, it was dissolved in a liquid solvent to obtain extracts. The lyophilized pulp was dissolved (1:10 *w*/*v*) in either distilled water or ethyl alcohol (70%) or a mixture of both (1:1) and homogenized for 3 min using a homogenizer. The homogenate was then sealed and kept at 40 °C for a duration of 4 h with intermittent shaking. Following this period, the homogenate was subjected to centrifugation, and the resulting supernatant was filtered through filter paper. The resulting extract was stored in a container at 4 °C. Water (W) or alcoholic (A) extracts or a mixture of both (W:A) prepared in this way were used to evaluate the content of polyphenols (TPC) and antioxidant activity.

### 2.2. Determination of the Antioxidant Potential of Dragon Fruit Extract

#### 2.2.1. Total Polyphenol Content (TPC) Determination

The determination of the total phenolic compound content followed the method developed by Singleton et al. [23]. The Folin–Ciocalteu reagent was first diluted with distilled water (1:5). Subsequently, 0.4 mL of this diluted reagent was added to the extract (0.1 mL) along with 0.1 mL of distilled water. After 3 min, Na_2_CO_3_ solution (10%; 2.5 mL) was added. The resulting samples were mixed and then kept in darkness for 30 min at room temperature. Following this time, the absorbance was measured at a wavelength of 725 nm. The concentration of phenolic compounds was determined using a standard curve available for gallic acid. The polyphenolic compound content was expressed in milligrams per milliliter of extract as gallic acid equivalent (GAE) [mg GAE/mL].

#### 2.2.2. Antioxidant Activity Determination

##### Antiradical Activity

The radical-scavenging activity was determined with commonly used methods: ABTS^•+^ scavenging activity (ABTS) [24] and DPPH scavenging activity (DPPH) [25]. The results are as follows:

Radical-scavenging activity (%) = (1 − A_2_/A_1_) × 100 (1)

where A_1_ is the absorbance of the control sample, and A_2_ is the absorbance of the sample.

##### Reducing Power

The reduction power (RP) was determined according to the Oyaizu method [26] which describes the ability of tested substances to reduce Fe^3+^ to Fe^2+^. The absorbance of samples was measured at a wavelength of 700 nm.

### 2.3. Development of Baked Meat Products

The fresh raw meat was pork shoulder, precrushed with a knife, and cured (1.8% addition of curing salt, 24 h, 4 °C). The meat was then ground using a meat grinder with a mesh diameter of 4 mm. Salt, pepper, garlic, and water (Table 1) were added to the meat stuffing. Additionally, dragon fruit pulp was added at varying concentrations of 0.5%, 1%, 2.5%, and 4%. This yielded the respective research variants: K (control, without dragon fruit) and P0.5, P1.5, P2.5, and P4. Then aluminum molds were filled with the meat stuffing, each containing 180 g, and baked at 180 °C for 45 min. After cooling, all pork meat products were placed in a tight foil package. They were stored in a refrigerator at 4 °C and analyzed immediately after production (1 day) as well as after 7, 14, and 21 days.

### 2.4. Proximate Composition

The chemical composition of the pork meat products was validated by various methods. The water content was determined through a drying process, while the total fat and total protein content were verified using near-infrared transmission spectrometry and calibrated on artificial neural networks [27] using FoodScan Lab (FOSS, Hillerød, Denmark). Carbohydrate content was determined by differences from the mentioned chemical compounds [18]. The calorific value of the product (kcal) was calculated in relation to 100 g of the sample, assuming that 1 g of fat provides 9 kcal, 1 g of protein provides 4 kcal, and carbohydrates contribute 4 kcal [28]. The ash content was determined according to PN-ISO 936:2000 [29]. The analysis was carried out immediately after production (day 1).

### 2.5. Mineral Content

The content of selected minerals (calcium, magnesium, potassium, and iron) was determined by flame atomic absorption spectrometry (FAAS), and the phosphorus content was determined using the spectrophotometric method, following the procedure described by Grdeń and Sołowiej [30].

### 2.6. pH, Oxidation-Reduction Potential, and Water Activity of Baked Pork Meat Product

The pH value was measured potentiometrically. Initially, the homogenates were obtained by homogenizing the product with distilled water at a ratio of 1:9 for 60 s using the Ultra-Turrax T25 Basic (IKA, Staufen, Germany). The pH measurement was carried out using a pH meter (Elmetron, Poland) equipped with a pH electrode (ERH-111, Hydromet, Poland).

Oxidation-reduction potential (ORP) was determined following the method outlined by Nam and Ahn [31] using a digital pH-meter with temperature compensation (CPC-501; Elmetron) set to the millivolt scale and equipped with a platinum redox electrode (ERPt-13; Hydromet).

The LabMaster-aw analyzer (NOVASINA AG, Lachen, Switzerland) was used to measure water activity (aw), according to the manufacturer’s instructions.

### 2.7. Thiobarbituric Acid Reactive Substances (TBARS) Values

The extent of lipid oxidation in the product was assessed by measuring the amount of TBA-reactive substances at four different time points: 0, 7, 14, and 21 days of storage at 4 °C. This assessment was conducted according to the procedure described by Pikul et al. [32].

### 2.8. Fatty Acids Profile

Gas chromatography was used to determine the profile of fatty acids after the conversion of fats to fatty acid methyl esters [33]. Lipid extraction from the samples followed the method established by Folch et al. [34]. The gas chromatography analysis was performed using a chromatograph (Varian 450-GC, Walnut Creek, CA, USA) equipped with a capillary column (Select Biodiesel for FAME, Varian, Palo Alto, CA, USA, 30 m × 0.32 mm × 0.25 µm layer thickness). The injector and detector temperatures were 250 °C and 300 °C, respectively. After injection, the column temperature was programmed to increase to 200 °C for 10 min, then increased to 240 °C at a rate of 3 °C/min, and then held at the final temperature for 4 min. Helium (3 mL/min) was used as the carrier gas. Fatty acid quantities were calculated from the chromatograms and an internal standard involving FAME and were: C6:0 (caproic acid), C8:0 (caprylic acid), C10:0 (capric acid), C12:0 (lauric acid), C14:0 (myristic acid), C14:1n5 (oleomyristic acid), C15:0 (pentadecanoic acid), C16:0 (palmitic acid), C16:1n7 (palmitoleic acid), C17:0 (margaric acid), C17:1n7 (cis-9-heptadecenoic acid), C18:0 (stearic acid), C18:1n9C (oleic acid), C18:1n9t (elaidic acid), C18:2n6c (linoleic acid), C18:2n6t (linolelaidonic acid), C18:3n3 (alpha) (alpha-linolenic acid), C20:0 (arachidonic acid), C20:1n 15 (cis-5-eicosenoic acid), C20:1n9 (cis-11-eicosenoic acid), C20:2n6 (cis-11,14-eicosadienoic acid), C21:0 (heneicosanoic acid), C20:4n6 (arachidonic acid), C20:5n3 (cis-5,8,11,14,17-eicosapentaenoic ac-id), C22:0 (behenic acid), C22:1n9 (erucic acid), C22:6n3 (cis-13,16-docosadienoic acid), and C24:1n9 (nervonic acid).

### 2.9. Antioxidant Activity of Pork Meat Product Extract

The pork meat product extracts were obtained by homogenizing 10 g of the product with 100 mL of distilled water using an IKA homogenizer (T25 Basic Ultra Turrax; IKA, Germany) at a speed of 10.5 rpm/min for 2 min. Following homogenization, the homogenate underwent centrifugation (5000× *g*; 10 min, 4 °C). The resulting supernatant was then filtered through filter paper and kept under refrigeration conditions for a maximum of 24 h for subsequent use. Antiradical activity against ABTS and DPPH cation radicals, as well as the ability to reduce iron ions (RP), were determined according to the method described in Section 2.2.2.

### 2.10. Statistical Analysis

Based on a two-factor analysis of variance (time, treatment), homogeneous groups were separated using Tukey’s test. Differences were considered statistically significant at *p* < 0.05. All data are presented as mean values ± standard error. For multivariate analyses, hierarchical clustering analysis (HCA) was performed to highlight differences between study variants (K, P4, P2.5, P1.5, and P0.5). The dataset was processed using Ward’s method of linkage with squared Euclidean distance, and the samples with maximum similarities were presented as a common cluster in the dendrogram. To identify the primary factors contributing most to the differentiation between variants, principal component analysis (PCA) was applied. In this study, multivariate analyses were performed based on the results obtained immediately after manufacturing (day 1). The collected data were analyzed using Statistica version 13.3 software. The data were not standardized before measurement. All measurements were performed on three independent series.

## 3. Results

### 3.1. Evaluation of the Antioxidant Potential of Dragon Fruit Pulp Extracts

As shown in Table 2, the highest level of total polyphenol content was detected in the alcohol extract from lyophilized dragon fruit pulp, while the lowest polyphenol content was found in the aqueous extract (*p* < 0.05). The aqueous extract demonstrated a significantly higher ability to neutralize the synthetic ABTS radical in comparison to other solvents. Simultaneously, it exhibited the lowest antiradical properties in the DPPH test. The values of the reduction power did not differ significantly among the test variants (*p* > 0.05).

### 3.2. Pork Meat Product Evaluation

The nutritional profile of pork meat products fortified with lyophilized dragon fruit pulp is presented in Table 2. The use of plant-based fortification in pork meat products resulted in a dose-dependent increase in the content of fat and carbohydrates in the product, although these differences were not statistically significant (*p* > 0.05). An exception to this trend was observed in the variant with a 4% addition of lyophilizate (P4), which exhibited a significantly increased (*p* < 0.05) share of fat and carbohydrates compared to the control (K). Despite the absence of significant differences in the content of basic nutrients among the analyzed variants, the use of lyophilized dragon fruit pulp led to a significant increase in the calorific value of the products, which turned out to be the highest in variant P4 (*p* < 0.05). The applied technological treatment did not affect the levels of water and ash content in the analyzed variants (*p* > 0.05).

The addition of the pulp from *H. undatus* to the pork meat product affected the content of selected chemical elements (Table 3). There was a dose-dependent increase in calcium and magnesium content (*p* < 0.05). At the same time, the applied technological treatment was associated with a decrease in the phosphorus content in products with dragon fruit pulp (P0.5, P1.5, P2.5, and P4) compared to the control variant (K). Potassium content remained unchanged in variants K, P4, and P0.5 (*p* > 0.05) and exhibited a slight increase in samples with 2.5% (P2.5) and 1.5% (P1.5) lyophilizate additions (*p* < 0.05). At the same time, these variants (P2.5 and P1.5) demonstrated a lower level of iron content compared to the control (K) (*p* < 0.05).

The changes in typical physicochemical parameters, reflecting the extent of quality changes in pork meat products during cold storage, are presented in Table 4. An initial reduction in the pH value of products with the addition of lyophilized dragon fruit pulp was observed immediately after preparation, and this trend continued throughout the storage period (21 days). The water activity values in the produced pork meat products were similar among the variants (*p* > 0.05), except for the variant with the highest lyophilizate dosage (P4), which showed a significantly higher value of aw on the 14th and 21st days of storage.

The ORP index is a parameter for determining the oxidative state of the meat matrix, with a higher ORP indicating a lower ability to neutralize oxidation factors. Right after the production of meat products, the ORP values did not differentiate between the analyzed variants. However, over time, a reduction in the ORP value was observed in variants with a lower dose of lyophilizate, specifically 0.5% (P0.5) after 7 and 21 days of cold storage and 1.5% (P1.5) on the 14th and 21st days of storage when compared to the control variants. Another indicator for assessing the degree of changes occurring in pork meat products is TBARS, which measures the degree of lipid peroxidation. In general, higher TBARS values were observed in products enriched with lyophilized dragon fruit pulp throughout the storage period. The only exception was the variant with the lowest dose, i.e., 0.5% lyophilizate addition (P0.5). The magnitude of the difference in TBARS levels is directly proportional to the dose used. In particular, the P4 variant exhibited an increased content of secondary fat oxidation products compared to the control throughout the storage period (*p* < 0.05) (Table 4).

The addition of dragon fruit pulp lyophilizate changed the fatty acid content of the pork meat products. The changes in individual fatty acids among the test variants are presented in Table 5. The introduction of the fortification treatment improved the fatty acid profile, resulting in an increase in the content of myristic acid, palmitic acid, arachidonic acid, and cis-5,8,11,14,17-eicosapentaenoic acid. In addition, the variant with a 4% dose showed an increase in the content of caprylic acid, palmitoleic acid, stearic acid, oleic acid + elaidic acid, linoleic acid + linolelaidonic acid, cis-5-eicosenoic acid, and cis-11-eicosenoic acid. Table 6, on the other hand, presents the fatty acid profile of pork meat products immediately after production (day 1).

The impact of the applied technological treatment, involving the fortification of the pork meat product with lyophilized dragon fruit pulp, was examined. By the end of the research period, products with a 4% addition of lyophilizate exhibited a higher content of SFA, MUFA, and PUFA. They also had a higher content of OMEGA fatty acids compared to the control group. In contrast, the test variant with the lowest lyophilizate dose was characterized by a reduced content of fatty acids from the SFA, MUFA, and PUFA groups, as well as OMEGA acid.

The antioxidant activity of extracts from enriched pork meat products during 21 days of storage is presented in Table 5. Observations were made regarding the effect of the storage period and the doses of plant additive application on the antiradical activity of pork meat products. The highest antiradical capacity (relative to ABTS and DPPH) was found in the tests on the 14th day of refrigerated storage, while the highest RP value was recorded on the 7th day of the analysis. Taking into account individual tests of antioxidant activity, the test variants with 4% (P4) and 2.5% (P2.5) addition of lyophilizate exhibited a higher ability to neutralize the ABTS radical compared to the control, and this trend continued throughout the research period (21 days). On the other hand, the antiradical activity against ABTS radical cations of the P1.5 and P0.5 variants was lower compared to the variant without the plant additive (K). Similarly, test variants with an increased proportion of lyophilizate (i.e., P4 and P2.5) demonstrated higher activity against DPPH up to the 14th day of refrigerated storage. Meanwhile, no significant differences in antiradical activity were noted for the other test variants (P1.5 and P0.5). In the last research period (i.e., 21 days), variants P1.5 and P0.5 exhibited reduced antiradical capacity in relation to DPPH. At the same time, these variants consistently exhibited a higher RP value compared to the control throughout the entire cold storage period (*p* < 0.05) (Table 7).

**Figure 1:**
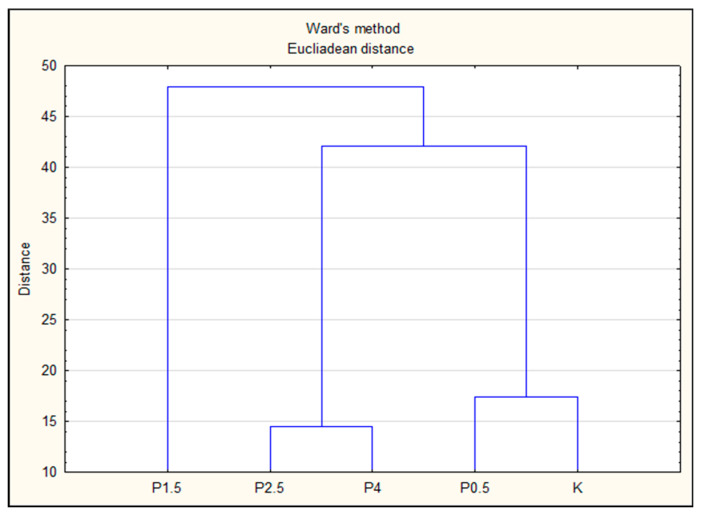
Dendrogram resulting from Ward’s method of hierarchical cluster analysis showing trends between research variants.

In this study, the method of hierarchical grouping was used to determine the extent of difference between pork meat product samples produced by different production methods. Based on the results, the test variants were grouped according to their antioxidant trends (ABTS, DPPH, and RP), fatty acid profile (SFA, MUFA, PUFA, OMEGA), and basic physicochemical characteristics (pH, ORP, aw, TBARS) immediately after production (day 1). Hierarchical Cluster Analysis (HCA) was used in this study to calculate multivariate Euclidean distances between observations (K, P4, P2.5, P1.5, and P0.5). Using a stepwise algorithm (Ward’s linkage criterion), observations with similar behavior in the initial variables were grouped together. The results were presented graphically in the cluster tree (Figure 1).

Groups of observations exhibiting similar behavior were grouped into clusters. The derived dendrogram made it possible to distinguish three distinct groups. Cluster 1 included the control variant and the variant with the lowest dose of lyophilizate (P0.5), indicating that this procedure had little effect on the result differentiation. Cluster 2 included variants with higher doses of lyophilized dragon fruit pulp, i.e., 2.5% (P2.5) and 4% (P4), which confirms the impact of higher doses of smog fruit pulp on the quality and nutritional potential of pork meat products. Conversely, the research variant P1.5 (with a 1.5% plant additive) was grouped completely separately as cluster 3.

**Figure 2:**
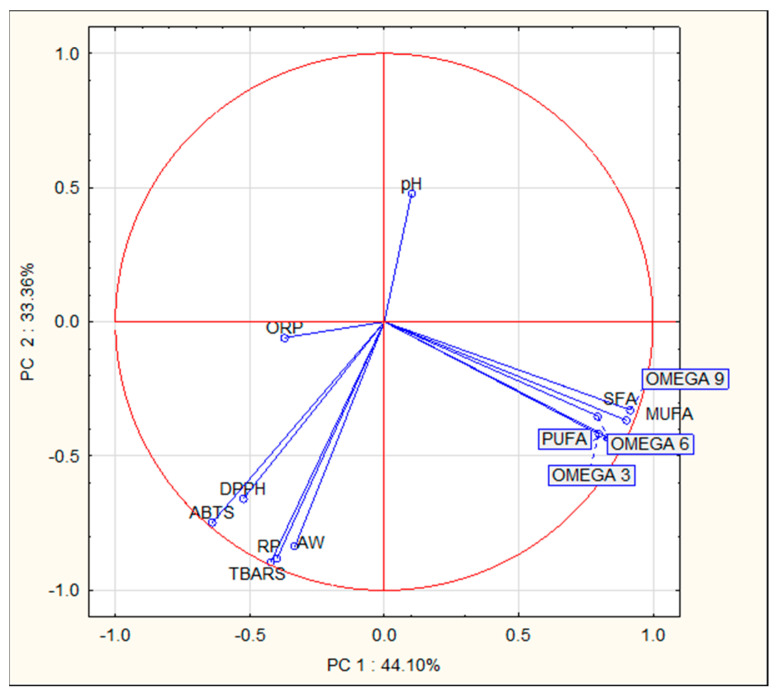
Graph of the configuration of points representing variables in a system of the first two factorial axes (principal components PC1 and PC2).

Principal component analysis (PCA) was used in this study to determine the most influential factors of variation contributing to the differences observed between the variants. Figure 1 presents the analyzed features of the products defined by the first two components (PC1 and PC2) along with their correlation (Figure 2). These two principal components collectively account for 77.46% of the total variance, with 44.10% of the variance in general characteristics explained by the first principal component (PC1).

The results of the PCA (Figure 2) and the correlation between variables showed that certain components of the pork meat product were associated with changes in other variables. Right after production, the content of fatty acids (defined as SFA, MUFA, PUFA, and OMEGA acids (−3, −6, −9)) constituted a distinct factor affecting the diversity of the analyzed variants of pork meat products, indicating a strong interrelationship between these two variables during the experiment. Additionally, fatty acids exhibited negative correlations with product quality features such as TBARS, aw, and the antioxidant activity (ABTS, DPPH, and RP) of extracts from pork meat products. The second principal component, explaining 33.36% of the cumulative variance, displayed a high correlation only with pH.

## 4. Discussion

In this study, extracts from *H. undatus* pulp were analyzed, indicating a higher level of antiradical activity when an alcoholic solvent was used. Similarly, the obtained extracts were characterized by a strong ability to scavenge DPPH radicals but exhibited weaker activity using ABTS. Thus, it seems that the use of water at the stage of preparing the meat stuffing as a solvent for BACs derived from dragon fruit appears to be the optimal choice because it does not contain residues, thus making it safe for consumption [35]. In addition, the analysis of *H. undatus* pulp confirmed the presence of carbohydrates, tannins, saponins, anthocyanins, quinones, glycosides, terpenoids, triterpenoids, phenols, acids, and steroids in the aqueous extract [36]. These compounds may significantly contribute to the prohealth properties of the meat product fortified with lyophilized dragon fruit pulp.

While adding plant additives to products of animal origin for the addition of the expected nutritional and functional properties, it is important to ensure that they do not have a negative impact on its basic parameters related to consumer acceptability (color, smell), as well as food quality and safety during the entire shelf life. The common determinant of these features is physicochemical changes occurring during storage, such as changes in pH, aw, or oxidation indexes (TBARS). In this study, the effect of lyophilized dragon fruit on the water activity and ORP of meat products was generally not confirmed. Only the addition of 4% lyophilizate resulted in increased aw from the 14th day of refrigerated storage. On the other hand, lower doses of lyophilizate (0.5% and 1.5%) contributed to a decrease in the ORP values during this time, compared to the control variant (K). The effect of the applied technological treatment on the reduction of the active acidity of the products during 21 days, regardless of the dose of the lyophilizate, was also demonstrated (Table 3). These observations presented in this study are consistent with previous findings reporting a decrease in the pH value of chicken meat [16] or sausage beef [18] after adding dried dragon fruit peel. Additionally, Bellicci et al. [21] showed the effect of the addition of dragon fruit at a dose of 1000 mg/kg which led to a decrease in pH in pork patties with a total replacement of animal fat. However, they did not find significant differences between the variants after 18 days of refrigerated storage at 2°C. The acidity in dragon fruit pulp mainly results from organic acids present in the fruits and thus can also significantly affect the pH of the pork meat product. Arivalagan et al. [37] showed that the flesh of the dragon fruit has a slightly acidic pH, which ranged from 4.8 to 5.40 (depending on the variety). Acids are known to be involved in various functions in the human body, including growth and maturation. Organic acids, by lowering the pH, not only influence organoleptic properties but also enhance shelf life, stability, and microbiological safety [37], thus playing an important role in the development of a new functional product that is safe for the consumer. In addition to organic acids, dragon fruit pulp is rich in various compounds with antioxidant properties. However, in this study, an increase in TBARS was noted in products with the addition of pulp lyophilizate, which could be due to lipid oxidation and the production of volatile metabolites under aerobic conditions. Previous studies have reported lower TBARS values in beef sausage with dried *H. polyrhizus* peel [18] or chicken nuggets with dried *H. undatus* peel [16] extracts compared to meat products without these extracts. However, in this study, the pulp with seeds (rich in fats) was used, not the skins, which may have a richer composition with a higher proportion of pro-oxidative components. In addition, as suggested by other researchers [38,39], lipid oxidation leads to PUFA breakdown and the secondary breakdown of various compounds. Since a large amount of aldehydes are formed during lipid oxidation, these compounds are reasonable candidates for reaction with thiobarbituric acid (TBARS). Thus, the higher TBARS values in pork meat products may be due to the higher content of unsaturated fatty acids, particularly those susceptible to oxidation processes, as indicated by the analysis of the fatty acid profile presented in Table 4. However, it should be noted that the TBARS values remained relatively low during the entire storage period, not exceeding 2.0 mg MDA/kg, indicating low production of potentially harmful compounds that could threaten consumer health [40].

The nutritional value of food products depends on their chemical composition. The use of lyophilized dragon fruit pulp at the stage of production of meat products generally did not significantly affect the basic chemical composition. of the products. This aligns with the results reported by Manihuruk et al. [18], who found that the inclusion of *H. polyrhizus* skin extracts in beef sausage had no significant impact on its water, ash, fat, or carbohydrate content. Similar observations were made by Madane et al. [16], who evaluated the approximate composition of chicken nuggets after adding powdered skin of *H. undatus*. The authors indicated that moisture, protein, fat, and ash content were not significantly different between the control group and the vegetable-enriched group, although the treated chicken nuggets had a slightly higher moisture content, possibly due to water absorption by the fruit powder during emulsion preparation. In this study, it was noted that the water content in lyophilized products was slightly lower than in the control group. However, it should be noted that dragon fruit peels contain a significant amount of fiber, which can contribute to water retention in the product, which may explain these differences. In addition, an increased proportion of carbohydrates was observed in this study, especially in the variant with the highest dose of lyophilizate (4%). Indeed, the white-fleshed dragon fruit contains a much higher total sugar content, including glucose and fructose, compared to *H. polyrhizus* fruit. In addition, among the carbohydrates in dragon fruit, oligosaccharides deserve attention. According to research presented by Wichienchot et al. [41], dragon fruit oligosaccharides are resistant to hydrolysis by artificial human gastric juice and human α-amylase, with maximum hydrolysis rates of 4.04% and 34.88%, respectively. It was found that oligosaccharides are also able to stimulate the growth of lactobacilli and bifidobacteria, which may have additional positive health effects after their consumption. While the increase in carbohydrate content in pork meat products due to the inclusion of lyophilized dragon fruit pulp was associated with an increase in caloric content, the lower sugar content of dragon fruit compared to commonly consumed fruits makes it less caloric and thus may be a good choice for enhancing health benefits.

Dragon fruit is also rich in minerals. It contains large amounts of phosphorus and calcium, which help to strengthen bones, play an important role in the formation of tissues, and create healthy teeth [6,11]. In addition, *H. polyrhizus* fruit has a high iron content, which helps increase hemoglobin and red blood cell levels in pregnant women [42], and its phosphorus content aids in tissue formation [11]. According to the research conducted by Arivalagan et al. [37], who assessed the nutritional value of *Hylocereus* spp., the consumption of 100 g of fresh dragon fruit covers a significant portion of the recommended daily intake (RDA) for several essential minerals. For instance, it provides about 11.4% of the RDA of magnesium; 5.7% RDA of copper; 5.4% RDA of calcium; 4.9% RDA of Phosphorus; 4.7% RDA of iron; 4.2% RDA of potassium, 2.5% RDA of manganese, and 2.4% RDA of zinc (averaged percentages) [37]. In this study, the addition of lyophilized pulp from *H. undatus* to the pork meat products led to an increase in calcium and magnesium content (*p* < 0.05), while it was associated with a decrease in phosphorus levels in products with dragon fruit pulp (P0.5, P1.5, P2.5, and P4) compared to the control group (K). Only the addition of 4% lyophilizate resulted in a noticeable increase in iron content in the pork meat product. Our research also highlighted a significant effect of dragon fruit pulp addition on the production of fatty acids. The fatty acid profiles were primarily characterized by MUFA (43.69% to 44.47% of total methyl esters), followed by SFA (from 44.55% to 46.01% of total methyl esters), and finally PUFA (from 5.17% to 6.22% of total methyl esters). In particular, the addition of 4% lyophilizate led to an increase in the content of fatty acids, including PUFA, which are crucial for maintaining the efficiency of the brain as they promote the proper development of the nervous system and its functionality. PUFA fatty acids have a positive effect on the structure of the skin, have a moisturizing, rebuilding, and regenerating effect, and improve the condition of acne skin. Lyophilized dragon fruit variants also exhibited higher proportions of OMEGA acids, including OMEGA-3, which are not naturally produced by the body and therefore need to be supplied through diet or appropriate supplementation. OMEGA-3 acids are important for the production of tissue hormones, including sex hormones, and are integral components of biological membranes, affecting brain and nervous system function [43,44]. To evaluate the nutritional quality of the lipids, the ratio *n* − 6/*n* − 3 was determined (Table 5). The values obtained for this ratio exceeded the maximum limit recommended by the FAO [45], which should ideally be less than 4. In this regard, Bellucio et al. [21] also reported *n* − 6/*n* − 3 ratios exceeding 4 (the maximum limit recommended) in pork patties containing dragon fruit, although the authors of that study replaced animal fat with an emulsion of tiger nut oil. Ariffin et al. [46], through oil extraction from dragon fruit seeds and the evaluation of two pitaya varieties (*H. undatus* and *H. polyrhizus*), identified a significant percentage of essential fatty acids, including linoleic acid and linolenic acid, along with two isomers of oleic acid). Adequate intake of linoleic acid contributes to the reduction of low-density lipoprotein (LDL-C) cholesterol levels in the blood, which in turn significantly reduces the risk of cardiovascular disease [47]. The essential fatty acids, namely linoleic acid and linolenic acid, constitute a significant percentage of the unsaturated fatty acids in the seed oil extract. Both varieties of pitaya contain about 50% of essential fatty acids (C18:2 (48%) and C18:3 (1.5%) [46], which probably influenced the outcomes observed in this study.

The antioxidant potential of pork meat products was also considered in this experiment. The antioxidant activity of extracts from pork meat products fortified with dragon fruit pulp showed the effect of the applied treatment on the antioxidant potential of the meat matrix to counteract oxidative factors. The obtained meat extracts showed stronger anti-radical activity against ABTS compared to DPPH, and this activity intensified with higher lyophilizate doses. Additionally, variants of the plant supplement were also characterized by an altered ability to reduce iron ions during the 21-day storage period. Phenolic compounds in plants have previously been reported to be effective scavengers of free radicals. However, the available information regarding the specific contribution of individual phenolic compounds to the overall antioxidant capacity of *H. undatus* pulp compared to its peel is somewhat limited. For instance, Tang et al. [48] studied the main phenolic compounds in the peel of *H. undatus*. The authors showed that the main phenolic contributors to antioxidant capacities in dragon fruit peel seem to be caffeic acid > p-coumaric acid > syringic acid. At the same time, the authors indicated that chlorogenic acid, nicotiflorin, quercetin, ferulic acid, rutin, and isoquercitrin appeared to not contribute to the antioxidant activity for the ABTS, DPPH, and FRAP assays in *H. undatus* peel. In contrast, the pulp of this dragon fruit variety was found to be rich in chlorogenic acid, but no ferulic acid or sinapaldehyde were detected. At the same time, the presence of rutin, hesperidin (flavonoids), and ascorbic acid, which may contribute to the antioxidative effect, has been demonstrated [48]. However, it should be emphasized that individual phenolic compounds can interact antagonistically or synergistically with one another [49] as well as with other compounds. For example, Choo et al. [6] showed that the ascorbic acid contained in *H. undatus* can act synergistically with phenols in radical scavenging activity.

## 5. Conclusions

The study shows the presence of compounds in the flesh of *H. undatus* that have promising health benefits. In particular, the content of selected minerals and fatty acids increases in products with plant additives, which constitute additional value for these meat products. In particular, baked pork meat products fortified with 4% lyophilized dragon fruit pulp were characterized by increased nutritional value without any negative effects on the pork meat product. Therefore, the introduction of dragon fruit pulp in pork meat processing could contribute to improving the health of consumers.

## Figures and Tables

**Table 1:** Additives used for each variant of pork meat products.

	K	P4	P2.5	P1.5	P0.5
Pork shoulder [g]	1000	1000	1000	1000	1000
Curing salt [%, *w*/*w*]	1.8	1.8	1.8	1.8	1.8
Salt [%, *w*/*w*]	0.2	0.2	0.2	0.2	0.2
Pepper [%, *w*/*w*]	0.5	0.5	0.5	0.5	0.5
Garlic [%, *w*/*w*]	0.5	0.5	0.5	0.5	0.5
Water [%, *w*/*w*]	4.0	4.0	4.0	4.0	4.0
Lyophilized dragon fruit pulp [%, *w*/*w*]	-	0.5	1.5	2.5	4.0

**Table 2:** Comparison of the total polyphenol content and antioxidant effects of extracts from lyophilized dragon fruit pulp.

	*W*	*W*:*A*	*A*
TPC [mg gallic acid/mL]	0.104 ± 0.02 ^a^	0.203 ± 0.04 ^b^	0.292 ± 0.07 ^c^
ABTS [%]	89.47 ± 2.53 ^a^	2.70 ± 1.09 ^b^	17.55 ± 7.06 ^c^
DPPH [%]	14.83 ± 3.52 ^a^	60.08 ± 6.50 ^b^	28.04 ± 6.46 ^c^
RP [*A* = 700]	0.142 ± 0.01 ^a^	0.138 ± 0.006 ^a^	0.142 ± 0.01 ^a^

Explanatory notes: TPC—total phenolics content, W—water extract; W:A—water:alcoholic extract (1:1), A—alcoholic extract. ^a–c^ Within the same test, means followed by a common letter do not differ significantly (*p* < 0.05).

**Table 3:** Comparison of the antioxidant effects of extracts from lyophilized dragon fruit pulp after production (day 1).

	*K*	P4	P2.5	P1.5	P0.5
Fat [%]	9.42 ± 0.82 ^a^	12.08 ± 1.04 ^b^	11.79 ± 1.01 ^a^	11.01 ± 0.94 ^a^	11.79 ± 0.88 ^a^
Protein [%]	21.11 ± 0.50 ^a^	20.07 ± 0.49 ^a^	19.72 ± 0.47 ^b^	19.64 ± 0.44 ^b^	21.45 ± 0.50 ^a^
Water [%]	68.29 ± 2.77 ^a^	63.54 ± 2.65 ^a^	64.89 ± 2.63 ^a^	65.74 ± 2.65 ^a^	64.08 ± 2.59 ^a^
Carbohydrates [%]	1.19 ± 0.31 ^a^	4.31 ± 1.35 ^b^	3.6 ± 0.91 ^a^	3.28 ± 1.24 ^a^	2.68 ± 0.72 ^a^
kcal	173.94 ± 6.84 ^a^	206.24 ± 3.54 ^c^	199.39 ± 3.51 ^b^	192.09 ± 4.02 ^b^	202.63 ± 3.84 ^b^
Ash [%]	2.50 ± 0.37 ^a^	2.35 ± 0.34 ^a^	2.43 ± 0.36 ^a^	2.47 ± 0.36 ^a^	2.50 ± 0.37 ^a^
Phosphorus [g/kg]	1.90 ± 0.01 ^a^	1.76 ± 0.01 ^b^	1.78 ± 0.01 ^b^	1.79 ± 0.01 ^c^	1.78 ± 0.01b ^c^
Calcium [mg/kg]	82.3 ± 2.72 ^a^	188.33 ± 6.51 ^b^	141.33 ± 6.51 ^c^	112.00 ± 3.00 ^d^	108.67 ± 5.51 ^d^
Magnesium [mg/kg]	154.67 ± 7.51 ^a^	247.33 ± 8.50 ^b^	235.67 ± 1.15 ^b^	249.33 ± 1.45 ^c^	220.33 ± 0.58 ^d^
Potassium [mg/kg]	3023.33 ± 15.28 ^a^	3033.33 ± 35.12 ^a^	3213.33 ± 35.12 ^b^	3156.67 ± 5.7 ^c^	3000.00 ± 30.00 ^a^
Iron [mg/kg]	34.33 ± 0.65 ^a^	48.43 ± 1.78 ^b^	17.23 ± 0.35 ^c^	20.27 ± 0.50 ^d^	19.47 ± 0.15 ^c d^

Explanatory notes: ^a–d^ Within the same treatment, means followed by a common letter do not differ significantly (*p* < 0.05).

**Table 4:** Comparison of the antioxidant effects of extracts from lyophilized dragon fruit pulp during storage (4 °C).

Parameter	Time [day]	*K*	P4	P2.5	P1.5	P0.5
pH	1	6.26 ± 0.024 ^aA^	6.12 ± 0.010 ^bAC^	6.26 ± 0.053 ^aA^	6.16 ± 0.010 ^bA^	6.15 ± 0.003 ^bA^
	7	6.25 ± 0.013 ^aA^	6.09 ± 0.013^cAB^	6.15 ± 0.002 ^bB^	6.12 ± 0.009 ^bcB^	6.14 ± 0.043 ^bcA^
	14	6.21 ± 0.043 ^aA^	6.06 ± 0.022 ^bB^	6.07 ± 0.013 ^bC^	6.06 ± 0.015 ^bC^	6.05 ± 0.010 ^bB^
	21	6.23 ± 0.034 ^aA^	6.15 ± 0.030 ^bC^	6.12 ± 0.022 ^bBC^	5.83 ± 0.012 ^cD^	6.03 ± 0.010 ^dB^
a_w_	1	0.988 ± 0.002 ^aA^	0.992 ± 0.002 ^aA^	0.992 ± 0.04 ^aA^	0.993 ± 0.0006 ^aA^	0.991 ± 0.001 ^aA^
	7	0.984 ± 0.002 ^aB^	0.991 ± 0.002 ^aB^	0.971 ± 0.040 ^aA^	0.988 ± 0.002 ^aB^	0.987 ± 0.002 ^aB^
	14	0.984 ± 0.003 ^acB^	0.990 ± 0.004 ^bB^	0.989 ± 0.002 ^abA^	0.984 ± 0.001 ^abC^	0.982 ± 0.002 ^cC^
	21	0.966 ± 0.003 ^acC^	0.972 ± 0.004 ^bC^	0.971 ± 0.002 ^abA^	0.956 ± 0.001 ^abD^	0.964 ± 0.001 ^cD^
ORP	1	322.63 ± 11.60 ^aA^	321.03 ± 10.15 ^aA^	324.00 ± 11.20 ^aAB^	338.40 ± 10.05 ^aA^	320.63 ± 2.61 ^aA^
	7	334.08 ± 6.56 ^aA^	343.80 ± 7.70 ^aB^	328.98 ± 3.03 ^abA^	322.53 ±22.28 ^abA^	312.36 ± 2.71 ^bA^
	14	301.13 ± 4.43 ^acB^	330.03 ± 6.40 ^bAB^	314.78 ±2.18 ^abB^	288 ± 14.26 ^cB^	313.25 ± 6.45 ^abA^
	21	336.13 ± 4.73 ^aA^	337.88 ± 3.17 ^aB^	336.38 ± 2.50 ^aA^	299.85 ± 5.14 ^bB^	274.50 ± 7.23 ^cB^
TBARS	1	1.00 ± 0.03 ^aA^	1.30 ± 0.04 ^bA^	1.26 ± 0.04 ^bcA^	1.33 ± 0.06 ^bA^	1.13 ± 0.10 ^acA^
	7	1.06 ± 0.22 ^aA^	1.84 ± 0.27 ^bB^	1.50 ± 0.07 ^bB^	1.15 ± 0.03 ^aA^	1.02 ± 0.04 ^aA^
	14	0.48 ± 0.01 ^aB^	0.62 ± 0.06 ^bC^	0.57 ± 0.02 ^bC^	0.53 ± 0.12 ^bB^	0.48 ± 0.24 ^aB^
	21	0.46 ± 0.05 ^aB^	0.83 ± 0.09 ^bC^	0.84 ± 0.14 ^bD^	0.36 ± 0.05 ^aB^	0.33 ± 0.04 ^aB^

Explanatory notes: ^A–D^ Within the same treatment, means followed by a common letter do not differ significantly (*p* < 0.05); ^a–d^ at the same time, means followed by a common letter do not differ significantly (*p* < 0.05).

**Table 5:** Individual fatty acids [g/100 g] of baked pork meat product with lyophilized dragon fruit pulp after production (day 1).

Parameter	*K*	P4	P2.5	P1.5	P0.5
C6:0	0.002 ± 0.000 ^a^	ND	0.002 ± 0.000 ^a^	0.002 ± 0.000 ^a^	0.003 ± 0.000 ^b^
C8:0	0.003 ± 0.000 ^a^	0.008 ± 0.001 ^b^	0.003 ± 0.001 ^a^	0.002 ± 0.000 ^a^	0.002 ± 0.000 ^a^
C10:0	0.011 ± 0.000 ^a^	0.013 ± 0.001 ^a^	0.011 ± 0.000 ^a^	0.013 ± 0.001 ^a^	0.012 ± 0.001 ^a^
C12:0	0.010 ± 0.001 ^a^	0.011 ± 0.001 ^a^	0.010 ± 0.001 ^a^	0.011 ± 0.001 ^a^	0.010 ± 0.000 ^a^
C14:0	0.147 ± 0.001 ^a^	0.178 ± 0.000 ^c^	0.155 ± 0.000 ^b^	0.152 ± 0.003 ^b^	0.144 ± 0.003 ^a^
C14:1n5	0.002 ± 0.000 ^a^	0.002 ± 0.000 ^a^	0.002 ± 0.000 ^a^	0.002 ± 0.000 ^a^	0.002 ± 0.000 ^a^
C15:0	0.006 ± 0.001 ^a^	0.005 ± 0.000 ^a^	0.005 ± 0.000 ^a^	0.005 ± 0.000 ^a^	0.006 ± 0.001 ^a^
C16:0	2.525 ± 0.001 ^a^	2.987 ± 0.010 ^d^	2.588 ± 0.013 ^c^	2.581 ± 0.003 ^c^	2.428 ± 0.023 ^b^
C16:1n7	0.253 ± 0.001 ^a^	0.302 ± 0.001 ^d^	0.259 ± 0.001 ^ac^	0.262 ± 0.001 ^c^	0.244 ± 0.003 ^b^
C17:0	0.030 ± 0.001 ^a^	0.029 ± 0.000 ^ab^	0.025 ± 0.000 ^c^	0.029 ± 0.000 ^ab^	0.028 ± 0.001 ^b^
C17:1n7	0.025 ± 0.000 ^a^	0.023 ± 0.001 ^a^	0.018 ± 0.000 ^b^	0.026 ± 0.001 ^a^	0.025 ± 0.001 ^a^
C18:0	1.441 ± 0.001 ^a^	1.597 ± 0.007 ^d^	1.392 ± 0.006 ^b^	1.469 ± 0.013 ^a^	1.365 ± 0.002 ^b^
C18:1n9_C_ + C18:1n9t	3.776 ± 0.01 ^ac^	4.298 ± 0.080 ^d^	3.684 ± 0.015 ^bc^	3.922 ± 0.023 ^a^	3.605 ± 0.002 ^b^
C18:2n6_C_ + C18:2n6t	0.464 ± 0.001 ^a^	0.619 ± 0.001 ^e^	0.453 ± 0.001 ^d^	0.559 ± 0.001 ^c^	0.424 ± 0.000 ^b^
C18:3n3 (alpha)	0.016 ± 0.000 ^a^	0.029 ± 0.000 ^e^	0.018 ± 0.000 ^d^	0.022 ± 0.000 ^c^	0.014 ± 0.001 ^b^
C20:0	0.017 ± 0.001 ^a^	0.026 ± 0.001 ^b^	0.023 ± 0.001 ^b^	0.018 ± 0.001 ^a^	0.017 ± 0.001 ^a^
C20:1n15	0.051 ± 0.001 ^a^	0.076 ± 0.001 ^e^	0.047 ± 0.001 ^d^	0.057 ± 0.005 ^c^	0.044 ± 0.001 ^b^
C20:1n9	0.021 ± 0.000 ^a^	0.028 ± 0.000 ^d^	0.020 ± 0.001 ^ab^	0.024 ± 0.000 ^c^	0.019 ± 0.000 ^b^
C20:2n6	0.003 ± 0.000 ^ab^	0.003 ± 0.000 ^ab^	0.002 ± 0.000 ^b^	0.004 ± 0.000 ^a^	0.003 ± 0.001 ^ab^
C21:0	0.007 ± 0.001 ^a^	0.008 ± 0.001 ^a^	0.004 ± 0.000 ^b^	0.009 ± 0.001 ^a^	0.004 ± 0.000 ^b^
C20:4n6	0.003 ± 0.000 ^a^	0.004 ± 0.000 ^a^	0.003 ± 0.000 ^a^	0.004 ± 0.001 ^a^	0.003 ± 0.000 ^a^
C20:5n3	ND	0.003 ± 0.001 ^a^	0.005 ± 0.000 ^c^	0.003 ± 0.000 ^b^	0.004 ± 0.000 ^abc^
C22:0	0.010 ± 0.000 ^a^	0.010 ± 0.000 ^a^	0.013 ± 0.000 ^b^	0.011 ± 0.001 ^ab^	0.011 ± 0.001 ^ab^
C22:1n9	0.004 ± 0.00 ^a^	0.007 ± 0.002 ^a^	0.006 ± 0.000 ^a^	0.005 ± 0.001 ^a^	0.005 ± 0.001 ^a^
C22:6n3	0.026 ± 0.020 ^a^	0.012 ± 0.001 ^b^	ND	ND	0.012 ± 0.000 ^b^

Explanatory notes: ND—not detected. ^a–d^ Within the same treatment, means followed by a common letter do not differ significantly (*p* < 0.05).

**Table 6:** Fatty acid profile [g/100 g] of baked pork meat product with apple pomace after production (day 1).

	*K*	P4	P2.5	P1.5	P0.5
SFA	4.206 ± 0.002 ^a^	4.782 ± 0.016 ^d^	4.236 ± 0.028 ^ac^	4.299 ± 0.006 ^c^	4.033 ± 0.032 ^b^
MUFA	4.131 ± 0.013 ^a^	4.783 ± 0.011 ^e^	4.033 ± 0.011 ^d^	3.942 ± 0.002 ^c^	3.942 ± 0.002 ^b^
PUFA	0.498 ± 0.003 ^a^	0.670 ± 0.004 ^b^	0.486 ± 0.011 ^a^	0.601 ± 0.001 ^c^	0.453 ± 0.009 ^d^
OMEGA 3	0.029± 0.001 ^a^	0.043 ± 0.004 ^b^	0.029 ± 0.010 ^ab^	0.034 ± 0.000 ^b^	0.023 ± 0.007 ^a^
OMEGA 6	0.470 ± 0.002 ^a^	0.626 ± 0.004 ^e^	0.457 ± 0.000 ^d^	0.567 ± 0.000 ^c^	0.430 ± 0.002 ^b^
OMEGA 9	3.801 ± 0.012 ^a^	4.382 ± 0.011 ^e^	3.710 ± 0.013 ^d^	3.951 ± 0.021 ^c^	3.628 ± 0.001 ^b^
OMEGA 6/3	16.21 ± 0.72 ^a^	14.77 ± 1.15 ^a^	14.49 ± 2.54 ^a^	16.66 ± 0.02 ^a^	18.69 ± 4.91 ^a^

Explanatory notes: OMEGA 3—sum of fatty acid: C18:3n3(alpha), C20:3n3, C20:5n3, C22:6n3; OMEGA 6—sum of fatty acid: C18:2n6c, C18:3n6 (gamma), C20:2n6, C20:3n6, C20:4N6, C22:2n6; OMEGA 9—sum of fatty acid: C18:1n9c, C20:1n9, C22:1n9, C24:1n9; ^a–e^ Within the same treatment, means followed by a common letter do not differ significantly (*p* < 0.05).

**Table 7:** Antioxidant potential of baked pork meat product fortified by lyophilized dragon fruit pulp during storage (4 °C).

Antioxidant Test	Time [day]	*K*	P4	P2.5	P1.5	P0.5
ABTS [%]	1	33.84 ± 2.88 ^aA^	43.75 ± 1.62 ^bAC^	43.53 ± 0.94 ^bAC^	29.85 ± 2.94 ^aA^	31.22 ± 0.91 ^aA^
	7	38.00 ± 2.60 ^aA^	44.73 ± 2.17 ^bA^	46.56 ± 2.20 ^bA^	34.17 ± 1.30 ^cB^	32.55 ± 1.53 ^cB^
	14	41.68 ± 3.66 ^aB^	53.15 ± 1.30 ^bB^	55.80 ± 2.74 ^bB^	38.49 ± 1.15 ^aC^	39.17 ± 0.82 ^aC^
	21	34.10 ± 3.34 ^aA^	41.14 ± 0.99 ^bC^	40.28 ± 2.74 ^bC^	26.15 ± 1.58 ^cA^	23.62 ± 1.75 ^cD^
DPPH [%]	1	23.16 ± 1.46 ^aA^	28.08 ± 1.45 ^bcA^	31.31 ± 2.63 ^cA^	25.50 ± 1.62 ^abA^	23.88 ± 1.40 ^aA^
	7	23.85 ± 1.49 ^aA^	33.22 ± 2.50 ^bB^	32.39 ± 1.15 ^bAB^	26.54 ± 1.15 ^aAB^	24.46 ± 1.28 ^aA^
	14	38.00 ± 2.32 ^aB^	39.99 ± 2.04 ^aC^	35.86 ± 1.83 ^aB^	28.43 ± 1.50 ^bB^	28.03 ± 1.89 ^bB^
	21	28.03 ± 1.81 ^aA^	28.72 ± 1.14 ^bA^	22.41 ± 0.96 ^acC^	21.37 ± 0.41 ^acC^	20.57 ± 1.36 ^cC^
RP [A_700_]	1	0.404 ± 0.012 ^aA^	0.789 ± 0.012 ^bA^	0.653 ± 0.002 ^cA^	0.514 ± 0.008 ^dA^	0.404 ± 0.012 ^aA^
	7	0.668 ± 0.025 ^aB^	1.212 ± 0.010 ^bB^	1.190 ± 0.028 ^bB^	1.079 ± 0.048 ^bB^	1.026 ± 0.009 ^bB^
	14	0.490 ± 0.014 ^aC^	0.612 ± 0.017 ^bC^	0.473 ± 0.038 ^aC^	0.853 ± 0.010 ^cC^	0.705 ± 0.016 ^dC^
	21	0.499 ± 0.002 ^aC^	0.512 ± 0.017 ^aD^	0.473 ± 0.040 ^aC^	0.853 ± 0.010 ^bD^	0.611 ± 0.011 ^cD^

Explanatory notes: ^A–D^ Within the same treatment, means followed by a common letter do not differ significantly (*p* < 0.05); ^a–d^ at the same time, means followed by a common letter do not differ significantly (*p* < 0.05).

## Data Availability

Data is contained within the article.
